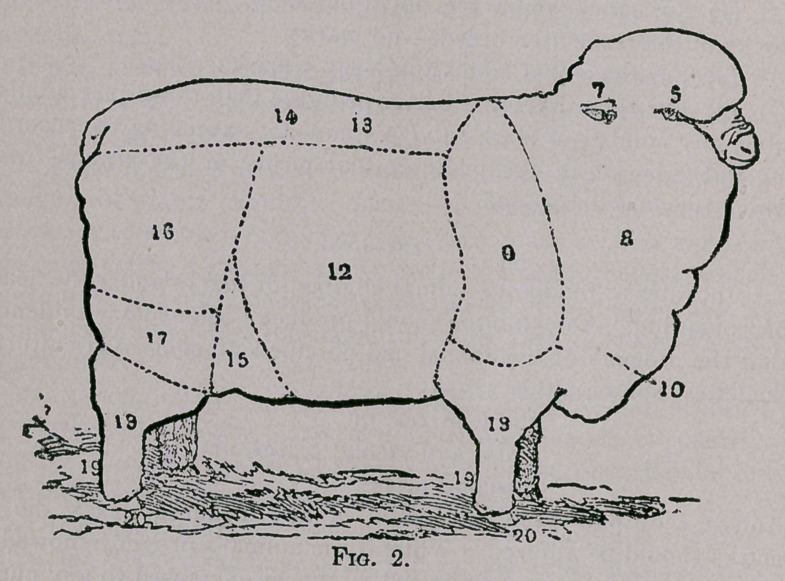# The Advantages of the Point System of Judging, and How It Should Be Initiated**The Agricultural Journal*, Cape Town, Cape of Good Hope, Africa.

**Published:** 1894-10

**Authors:** 


### THE ADVANTAGES OF THE POINT SYSTEM OF JUDGING, AND HOW IT SHOULD BE INITIATED.*

At a conference of delegates from Agricultural Societies, convened by the Department of Agriculture of New South Wales, it was pointed out that the aim and objects of the Agricultural Societies in that colony were to a large extent frustrated, and that a great part of the expenditure at many of the shows did not bring about any good to the agricultural community, owing to the following causes:—That the neighbouring Societies frequently clash in the date of holding their shows, instead of co-operating in this as well as in other matters of mutual importance; that prizes are given by Societies (1) for products and varieties of grain, &c., which are unsuitable for cultivation in their districts or for commercial purposes; (2) for breeds of fowls, &c., valuable for fancy purposes only; (3) for objects not connected with agriculture; (4) for exhibits of produce, especially grain, without making it compulsory for the successful competitor to show, if required, that his exhibit is a fair specimen of what has been produced by him on a commercial scale.

It was, however, agreed that the greatest difficulty with which the Societies had to contend was the lack of competent judges and the defective system of judging at present prevailing.

It was suggested as a remedy for this difficulty that a system of judging by points should be introduced which would render it unnecessary to employ more than one judge in any section, and the judge need not be a stranger to the district, a condition which had hitherto been generally adopted unless, as frequently happened, a lack of judges compelled the committee to request local men to act in that capacity.

Mr. Alexander Bruce, Chief Inspector of Stock, was accordingly requested to frame a system for judging animals by points, giving the relative value which should be attached to each point of the various animals usually exhibited at shows.

As the same difficulty besets Agricultural Societies in this country, Mr. Bruce's report is worthy of careful consideration. As, however, space does not permit of the whole of the report being inserted, we will confine ourselves to the system as regards sheep,

* *The Agricultural Journal*, Cape Town, Cape of Good Hope, Africa.

the want of competent judges in that class being most keenly felt throughout this Colony.

In submitting his report, Mr. Bruce mentions that although the points have been to some extent tested, and would, he is confident, produce more satisfactory decisions than are now as a rule given under the present system of judging, it is not to be expected that they are yet perfectly correct. Such a result can only be arrived at by practice and experience; but it is considered that the system will be found sufficiently accurate for all practical purposes.

The following explanation will enable the reader to more fully understand the system and the basis on which the values attached to the various points have been calculated.

PEDIGREE.

Combined with good shapes and a sound constitution and frame, a good pedigree is one of the most valuable points in stock, insuring as it does in a very high degree, when possessed by both sire and dam, success in breeding; for then, and then only, does the saying that "like begets like" hold good.

The marks for pedigree would be fairly awarded on some such basis as the following:—

(1) For stock which are not admissible in the stud or herd books of the respective breeds—no marks.

(2) For those just admissible—say 5 marks.

(3) For those having a better pedigree than those just admissible, any number of marks above 5 and not exceeding 20, according to the length of the pedigree and purity of the strains from which they are descended.

OFFSPRING.

In order to render an animal eligible for marks under the head of "offspring," the exhibitor should produce documentary evidence that the progeny of the animal had obtained at some Agricultural Societies' Shows, either say—

2 first prizes, or
3 first and second prizes, or
4 second prizes.

And if they had been successful in any of these cases, the 5 marks should be allowed. While if the animal's progeny showed a better record, the marks allotted might be increased to any number above 10 and not exceeding 20, according to the number of the prizes, and the standing of the shows and classes in which the prizes were won.

FIG. 1.

FIG. 2.

| No. | Description of Points and their Values of Medium Combing Ram. | Single Points. Aggregate 250. | Groups. Aggregate 100. | Divisions. Aggregate 100. |
|---|---|---|---|---|
| | I. BREEDING AND QUALITY. | | | |
| | 1. *Pedigree and Offspring.* | | | |
| 1. | "Pedigree."—According to standing in Stud Book, or as proved by certificates and declarations.................. | 20 | | |
| 2. | "Offspring."—To be viewed from the character of the offspring as shown by their success at Shows......... .. | 20 | 16 | |
| | 2. *General Appearance, Style and Character.* | | | |
| 3. | "Symmetrical form and proper complexion and covering.". | 10 | 4 | |
| | 3. *Head.* | | | |
| 4. | "Countenance."—The forehead should be broad and the countenance healthful.................................. | 5 | | 28 |
| 5. | "The Eyes."—Should be bright and placid and free from spots ................. .. ............................ | 3 | | |
| 6. | "The Muzzle,' &c.—The muzzle should be clean, the nostril expanded, and the nose white, wrinkly, and covered with short, furry, soft, velvety hair.................... | 5 | 8 | |
| 7. | "The Ears."—Should be white, soft, thick, wide apart, and partly covered with wool.... ........................ | 3 | | |
| 8. | "The Horns."—Should not be too close to the head and neck, nor standing out too widely, and should be free from black or dark streaks............................ | 4 | | |
| | II. FORM AND CONSTITUTION. | | | |
| | 4. *Fore Quarter.* | | | |
| 9. | "The Neck."—Should be short on the top, deep when viewed from the side, and long below, strongly set to the head and shoulders, towards which it should be becoming deeper.................................... ........ | 5 | | |
| 10. | "The Shoulders."—Should be broad and massive as to depth and breadth, very little, if at all, above the level of the back, and well placed................ ............. | 4 | 6 | |
| 11. | "The Chest."—Should be wide and deep.................. | 4 | | |
| 12. | "The Skin."—Should be thick, soft and pink.............. | 2 | | |
| | 5. *Middle.* | | | |
| 13. | "The Barrel."—Should be round and lengthy............. | 6 | | |
| 14. | "The Back."—Should be short, level, strong and straight ... ..................... ...... .............. | 5 | 6 | |
| 15. | "The Loin."—Should be broad and strong......... ....... | 4 | | 20 |
| | 6. *Hind Quarter.* | | | |
| 16. | "The Flank."—Should be deep and straight............... | 4 | | |
| 17. | "The Quarters."—Should be long and well filled up....... | 4 | 4 | |
| 18. | "The Thighs."—Should be long and broad.. ............ | 2 | | |
| | 7. *Legs, Feet, &c.* | | | |
| 19. | "The Legs."—The fore legs should be short, straight and well apart, and the hind legs should be set so as to give the hind parts a perpendicular appearance; while the bone should be heavy, but of fine texture............... | 5 | 4 | |
| 20. | "The Muscle."—Should be fine and firm... .............. | 2 | | |
| 21. | "The Hoofs."—Should be clear in color and well shaped... | 3 | | |
| | Carried forward................................. ..... | 120 | 48 | 48 |

| Description of Points and their Values of Medium Combing Ram. | Single Points. Aggregate 250. | Groups. Aggregate 100. | Divisions. Aggregate 100. |
|---|---|---|---|
| Brought forward ... ....... ............... ...... | 120 | 48 | 48 |
| II. FORM AND CONSTITUTION—*Continued.* | | | |
| 8. *Size.* | | | |
| 22. "Size."—According the class of sheep.... ............... | 5 | 2 | 2 |
| III. THE WOOL. | | | |
| 9. *Quantity.* | | | |
| 23. "Length of Staple."—According to division............... | 5 | | |
| 24. "Density."—Closeness and thickness all over, but especially on the top of the shoulder and back..................... | 30 | 22 | 22 |
| 25. "Evenness."—In length and density of fleece over the whole body, legs, belly, back and head... .. ....... ........ | 20 | | |
| 10. *Quality.* | | | |
| 26. "Brightness, including Lustre."—Denotes facility for taking delicate dyes.................................. | 5 | | |
| 27. "Softness."—Soft and silky to the touch, but elastic....... | 8 | 8 | |
| 28. "Crimp."—The regularity of the waves and trueness of the fibre. .. .......... ............................ | 7 | | |
| 29. "Freedom from Gare," *i. e.*, Kemp...................... | 5 | | |
| 30. "Fineness."—According to division.................. ....... | 17 | | |
| 31. "Freeness."—Denoting few noils in combing and including building up of staple.... .. ..................... ...... | 6 | 18 | 28 |
| 32. "Evenness."—In the quality of the fleece over the whole body, legs, belly, back and head....................... | 17 | | |
| 11. *Condition.* | | | |
| 33. "Quality of Yolk"...................... ........... .... | 3 | 2 | |
| 34. "Fluidity of Yolk"........... . ....................... | 2 | | |
| Aggregate numbers......................... ....... | 250 | 100 | 100 |

### ABSTRACT.

| | Single Points. | Groups. | Divisions. |
|---|---|---|---|
| Breeding and Quality................................ ........ | 70 | 28 | 28 |
| Form and Constitution..................................... | 54 | 22 | 22 |
| Wool....................... ................... .......... | 126 | 50 | 50 |
| Total Marks.......................... ......... | 250 | 100 | 100 |

THE AGGREGATE NUMBER OF POINTS.

In framing a scale of points, it is of course necessary that there should be a fixed aggregate of points; and in fixing this aggregate, care must be taken to do so at a number that will give the judges, when working under the point system, sufficient scope to mark the difference in merit of the point under consideration in the different animals. The following plates represent a Medium Combing Merino Ram and Ewe, the figures on the plates corresponding with the figures in the annexed table, in which the different points are described and the maximum values attached thereto set down.

The points and values for ewes are the same as for rams, with the following exceptions:—the marks for "muzzle" are, for ewes, 4 instead of 5. Those for "horns" are omitted, and 5 marks are added for "evenness of covering."

The above scale of points relates to medium combing sheep; by reducing the marks for "size" and "length of staple," and increasing those for "softness" and "fineness," the scale will be suitable for fine woolled sheep; and by increasing the marks for "length of staple" and "brightness," and reducing those for "softness" and "fineness," the scale will answer for strong woolled sheep.

## Figures and Tables

**Fig. 1. f1:**
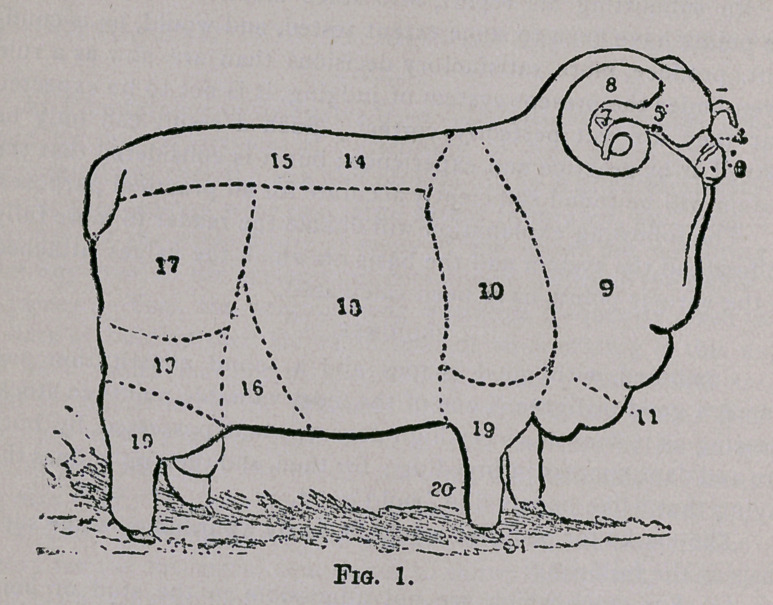


**Fig. 2. f2:**